# Supplementary material for: Time-series transcriptome analysis identified differentially expressed genes in broiler chicken infected with mixed Eimeria species
Source: Front Genet. 2022 Aug 8;13:886781. doi: 10.3389/fgene.2022.886781 (PMC9393255; doi:10.3389/fgene.2022.886781)
Supplement: Supplementary file 2 [file DataSheet1.ZIP › 4dpi_KEGG.Gsea.1625100298584/KEGG_AMINOACYL_TRNA_BIOSYNTHESIS.html]

Details for gene set KEGG\_AMINOACYL\_TRNA\_BIOSYNTHESIS[GSEA]

|  || Dataset | TMM\_4dpi\_gct\_format\_4dpi\_gct\_format.Class\_4dpi.cls #PC\_versus\_NC.Class\_4dpi.cls #PC\_versus\_NC\_repos |
| Phenotype | Class\_4dpi.cls#PC\_versus\_NC\_repos |
| Upregulated in class | 0 |
| GeneSet | KEGG\_AMINOACYL\_TRNA\_BIOSYNTHESIS |
| Enrichment Score (ES) | -0.49908292 |
| Normalized Enrichment Score (NES) | -1.467735 |
| Nominal p-value | 0.05457826 |
| FDR q-value | 0.23499925 |
| FWER p-Value | 0.967 |
Table: GSEA Results Summary

  

Fig 1: Enrichment plot: KEGG\_AMINOACYL\_TRNA\_BIOSYNTHESIS      
 Profile of the Running ES Score & Positions of GeneSet Members on the Rank Ordered List

  

| SYMBOL | TITLE | RANK IN GENE LIST | RANK METRIC SCORE | RUNNING ES | CORE ENRICHMENT || 1 | EARS2 | na | 1164 | 0.613 | -0.0108 | No |
| 2 | RARS2 | na | 2711 | 0.322 | -0.0944 | No |
| 3 | DARS2 | na | 3592 | 0.208 | -0.1385 | No |
| 4 | SEPSECS | na | 5202 | 0.061 | -0.2641 | No |
| 5 | MTFMT | na | 6096 | -0.014 | -0.3366 | No |
| 6 | NARS2 | na | 6570 | -0.052 | -0.3688 | No |
| 7 | PSTK | na | 7666 | -0.149 | -0.4391 | No |
| 8 | FARSA | na | 7935 | -0.173 | -0.4372 | No |
| 9 | LARS2 | na | 8678 | -0.248 | -0.4642 | Yes |
| 10 | SARS2 | na | 8762 | -0.257 | -0.4349 | Yes |
| 11 | HARS2 | na | 8938 | -0.278 | -0.4104 | Yes |
| 12 | AARS2 | na | 9015 | -0.284 | -0.3767 | Yes |
| 13 | FARSB | na | 9310 | -0.323 | -0.3557 | Yes |
| 14 | IARS2 | na | 9805 | -0.387 | -0.3424 | Yes |
| 15 | CARS2 | na | 10096 | -0.429 | -0.3063 | Yes |
| 16 | FARS2 | na | 10543 | -0.507 | -0.2721 | Yes |
| 17 | YARS2 | na | 10863 | -0.575 | -0.2177 | Yes |
| 18 | MARS2 | na | 10965 | -0.597 | -0.1421 | Yes |
| 19 | TARS2 | na | 11323 | -0.711 | -0.0718 | Yes |
| 20 | PARS2 | na | 11654 | -0.916 | 0.0297 | Yes |
Table: GSEA details [plain text format]

  

Fig 2: KEGG\_AMINOACYL\_TRNA\_BIOSYNTHESIS      
 Blue-Pink O' Gram in the Space of the Analyzed GeneSet

  

Fig 3: KEGG\_AMINOACYL\_TRNA\_BIOSYNTHESIS: Random ES distribution      
 Gene set null distribution of ES for **KEGG\_AMINOACYL\_TRNA\_BIOSYNTHESIS**
